# Editorial: Nanomedicine-Based Drug Delivery Systems: Recent Developments and Future Prospects

**DOI:** 10.3390/molecules28104138

**Published:** 2023-05-17

**Authors:** Faiyaz Shakeel

**Affiliations:** Department of Pharmaceutics, College of Pharmacy, King Saud University, Riyadh 11451, Saudi Arabia; fsahmad@ksu.edu.sa

Since the discovery of nanomedicine-based drug delivery carriers such as nanoparticles, liposomes, and self-nanoemulsifying drug delivery systems (SNEDDS), enormous progress has been achieved in the field of innovative active biomolecule drug delivery systems. The use of nanomedicines as drug delivery carriers has received lot of interest in recent years for the therapeutic targeting of specific cells. Biocompatibility, biodegradability, low toxicity, drug delivery efficiency, drug targeting efficiency, and improved solubility, bioavailability, and bioactivities are all advantages of these nanosized drug delivery carriers. Furthermore, these carriers can encapsulate a diverse range of active therapeutic biomolecules. These nanomedicine-based drug delivery carriers can also improve the pharmacokinetic and pharmacodynamic efficiency of active therapeutic biomolecules, allowing for a more sustained, targeted, and controlled drug delivery system. Various studies have recently shown progress in nanomedicine-based drug delivery systems for future therapeutic targeting. The aim of this Special Issue was to collect papers on recent advances, developments, and future prospects in the design, development, characterization, and biological evaluation of nanomedicine-based drug delivery systems of active therapeutic biomolecules.

This Special Issue starts with the paper by Shakeel et al. [1], who developed SNEDDS formulations of a bioactive compound, luteolin, in order to enhance its dissolution rate and hepatoprotective effects. Different SNEDDS formulations of luteolin were developed using an aqueous phase titration method, characterized physicochemically, and evaluated for in vitro drug release and hepatoprotective effects. The findings of this study indicate the potential of SNEDDS for the enhancement of the dissolution rate and hepatoprotective effects of luteolin.

Al-Joufi et al. [2] next enhanced the ocular bioavailability and antibacterial effects of ciprofloxacin using colloidal lipid-based carriers (liposomal drops) for the management of post-surgical infection. The liposomal drops of ciprofloxacin were characterized for various physicochemical parameters and evaluated for in vitro drug release, antibacterial effects, and pharmacokinetic studies. The results showed significant enhancement in the ocular bioavailability of ciprofloxacin using the liposomal drops compared to its commercial formulation.

Novel turmeric rhizome extract nanoparticles were developed and evaluated by Auychaipornlert et al. [3] in the next article. The prepared nanoparticles were characterized well with an optimized experimental design technique. The anticancer potential of nanoparticles was evaluated against the human hepatoma cells, HepG2. The proposed nanoparticles showed significant anticancer effects compared to pure curcumin. The results suggested the potential of nanoparticles of turmeric rhizome extract in the treatment of hepatocellular carcinoma. 

Thermosensitive liposomes were developed using the QbD approach by Dobo et al. [4] in the next article. Thermosenistive liposomal formulations were produced using different phospholipids and PEGylated lipids and optimized using the QbD approach. The findings showed that the application of different types and ratios of lipids influences the thermal properties of liposomes.

Kalam et al. [5] developed and evaluated noninvasive chitosan nanoparticles of tedizolid phosphate for the treatment of MRSA-related ocular and orbital infections. The release profile of the studied drug was sustained release from the chitosan nanoparticles compared to its aqueous suspension. The transcorneal flux and antibacterial effects of tedizolid phosphate-loaded chitosan nanoparticles were significant compared to its aqueous suspension. The findings of this work indicate the potential of chitosan nanoparticles in the treatment of MRSA ocular infections and related inflammatory conditions.

Rajput et al. [6] developed a liposome-loaded microneedle array patch of levonorgestrel for contraception. A levonorgestrel-loaded liposomal formulation was obtained using a solvent injection method, characterized for various physicochemical parameters and studied well. The findings of this study showed the better contraceptive effects of the levonorgestrel liposome-loaded microneedle array patch compared to the drug-loaded microneedle array patch.

The SNEDDS formulations of apremilast were developed and evaluated for the treatment of psoriatic arthritis in the next article [7]. Thermodynamically stable SNEDDS formulations were characterized physicochemically and then subjected to in vitro drug release and pharmacokinetics studies. The optimum formulation showed excellent physiochemical parameters and an excellent drug release profile. The significant enhancement in the drug release and bioavailability of apremilast SNEDDS was recorded compared to its suspension. The findings of this study suggest that apremilast SNEDDS is a possible alternative delivery system for apremilast. However, further studies exploring the major factors that influence the encapsulation efficiency and stability of apremilast SNEDDS were suggested. 

In another article, the antibacterial and cytotoxic properties of a novel fullerene derivative composed of C_60_ fullerenol and standard aminoglycoside antibiotic–gentamicin (C_60_ fullerenol–gentamicin conjugate) were evaluated [8]. In vitro assays suggested that the developed C_60_ fullerenol–gentamicin conjugate possessed the same antibacterial activity as standard gentamicin against various bacterial strains. The in vitro cytotoxicity assessment indicated that the fullerenol–gentamicin conjugate did not decrease the viability of normal human fibroblasts compared to control fibroblasts. The findings of this study suggested that the developed C_60_ fullerenol–gentamicin conjugate could have biomedical potential. 

Alshememry et al. [9] evaluated the successful utilization of the positively charged nanocrystals of tedizolid phosphate for topical ocular applications. The developed nanocrystals of tedizolid phosphate showed significant antibacterial activity against *B. subtilis*, *S. pneumonia, S. aureus*, and MRSA strains as compared to pure drug. Various pharmacokinetics parameters of nanocrystals were also increased significantly in rabbits compared to the pure drug in the ocular pharmacokinetic study. The nanocrystals of tedizolid phosphate were identified as a promising substitute for the ocular delivery of tedizolid phosphate, with better performance as compared to pure drug.

Two different PAMAM dendrimer generations, G4 and G5 dendrimers, were developed and evaluated in the next article [10]. Developed G4 and G5 dendrimers were characterized well and evaluated for in vitro drug release and cytotoxic effects in human lung adenocarcinoma cells. The findings of this study highlighted the potential anticancer effects of cationic G4 dendrimers as a targeting-sustained-release carrier for erlotinib.

Kenchegowda et al. [11] explore the potential of smart nanocarriers as an emerging platform for cancer therapy. In this exhaustive review, they focus on current advances made through the use of smart nanocarriers such as dendrimers, liposomes, mesoporous silica nanoparticles, quantum dots, micelles, superparamagnetic iron-oxide nanoparticles, gold nanoparticles, and carbon nanotubes. Various topics such as drug targeting, surface-decorated smart-nanocarriers, and stimuli-responsive cancer nanotherapeutics responding to temperature, enzyme, pH and redox stimuli have been covered in this review.

In the next review article, Kumar et al. [12] explore the potential of natural product-based nanomedicine for maintaining oral health. In their exhaustive review, the potential of natural products obtained from different sources for the prevention and treatment of dental diseases is discussed and summarized in the form of nanomedicines. 

In the next review, the application of the QbD approach is utilized in the robust product development of liposomal formulations [13]. This review discusses and summarizes the current practices that employ QbD in the robust development of liposomal-based nanopharmaceuticals. 

In recent years, there has been tremendous research on nanomedicine-based drug delivery systems. This Special Issue has brought together prominent scientists who have explored a diverse applications range of nanomedicine-based drug delivery systems. I believe that further clinical and toxicological studies on both animal and human models are still required to explore the complete potential and commercial exploitation of nanomedicine-based drug delivery systems. The diverse and critical perspectives within this Special Issue provide sufficient information on the development, characterization and evaluation of nanomedicine-based drug delivery systems.
